# *Burmoniscus kitadaitoensis* Nunomura, 2009 (Crustacea, Isopoda, Oniscidea) from southern Japan, a junior synonym of *B. meeusei* (Holthuis, 1947)

**DOI:** 10.3897/zookeys.386.6727

**Published:** 2014-03-06

**Authors:** Shigenori Karasawa, Kenshi Goto

**Affiliations:** 1 Faculty of Education, Fukuoka University of Education, 1-1 Akamabunkyo-machi, Munakata, Fukuoka, 811-4192 Japan; 2 Nanbu Agricultural Development Center, 517 Yamakawa, Haebaru-cho, Shimajiri-gun, Okinawa, 901-1115 Japan

**Keywords:** Kitadaitojima Island, mitochondrial DNA, nuclear DNA, Philosciidae, terrestrial isopods

## Abstract

Re-examination of the holotype of *Burmoniscus kitadaitoensis* Nunomura, 2009 from Kitadaitojima Island, southern Japan reveals that this species is a junior synonym of *B. meeusei* (Holthuis, 1947). Partial regions of mitochondrial COI, 12S and 16S rRNA genes, and nuclear 18S and 28S rRNA genes were detected for species identification in the future.

## Introduction

*Burmoniscus* Collinge, 1914 can be dominant in terrestrial isopod communities in subtropical forests of East Asia (Ma et al. 1991). Therefore clarifying the taxonomic status of *Burmoniscus* species is important to understanding species diversity of isopod communities in these habitats. Thirteen species in the genus *Burmoniscus* were reported from Japan (Nunomura 2011), but their taxonomic status is still confused (Karasawa and Honda 2012).

*Burmoniscus meeusei* (Holthuis, 1947) was first described as *Chaetophiloscia meeusei* Holthuis, 1947, based on specimens found from a greenhouse at the Royal Botanic Gardens, Kew, United Kingdom. Later, Taiti and Ferrara (1991) found this species in Hawaii and transferred it to the genus *Burmoniscus*. Since then this species has been found in Taiwan and Brazil (Kwon and Jeon 1993; Araujo et al. 1996). *Burmoniscus meeusei* can be distinguished from congeneric species by a small lobe on the inner margin of the apical part of the male pleopod 1 endopodite, a triangular distal part on the male pleopod 1 exopodite, and the round apex of the pleotelson (see Holthuis 1947). *Burmoniscus kitadaitoensis* Nunomura, 2009 was described from specimens collected on Kitaditojima Island, southern Japan, where it was supposed to be endemic because it was never reported from other areas (Nunomura 2011). In the original description, Nunomura (2009) compared the morphological features of *Burmoniscus kitadaitoensis* to those of *Burmoniscus okinawaensis* (Nunomura, 1986) and *Burmoniscus daitoensis* (Nunomura, 1986), and he concluded that *Burmoniscus kitadaitoensis* was an undescribed species. The figures of Nunomura (2009), however, show that the male pleopod 1 exopodite of *Burmoniscus kitadaitoesis* has a similar shape to that of *Burmoniscus meeusei* (e.g., Holthuis 1947). However, the small lobe of the male pleopod 1 endopodite is not illustrated in the figures.

The aim of this study is to examine the holotype of *Burmoniscus kitadaitoensis* and clarify its taxonomic status. Moreover, partial sequences of the mitochondrial COI, 12S and 16S rRNA genes, and nuclear 18S and 28S rRNA genes are detected for DNA markers of species identification.

## Materials and methods

### Sample collection

The holotype of *Burmoniscus kitadaitoensis* was deposited in Toyama Science Museum (male, TOYA-Cr 14899). We examined the holotype, but the specimen was dissected and in bad condition. However, we were able to observe some parts as follows: male pleopod 1 endo- and exopodites, pleopod 2 endo- and exopodites, male pereiopods 1 and 7, genital papilla, epimera of pereionite 7, and pleotelson. Four specimens were also collected from Kitadaitojima Island (type locality) and Amamioshima Island and were used for measurements of the co-ordinate of the noduli laterales and molecular analysis. The voucher specimens are deposited in the collection of Kitakyushu Museum of Natural History and Human History (KMNH-IvR), Kitakyushu, Fukuoka Prefecture, Japan.

## Morphology

The male pleopods 1 and 2, genital papilla, and male pereiopods 1 and 7 of the holotype, and the position of noduli laterales of specimens collected from Kitadaitojima Island were examined using a Nikon Eclipse E400 microscope (magnification of 40–400×). The epimera of pereionite 7 and pleotelson of the holotype were examined using an Olympus SZH-10 microscope (magnification of 7–64×). A color image was produced from multi-focused montage images using a digital microscope VHX-2000 (KEYENCE Corporation).

### Molecular analysis

The partial sequences of mitochondrial cytochrome oxidase subunit I (COI), mitochondrial 12S and 16S ribosomal RNA (rRNA) genes, and nuclear 18S and 28S rRNA genes were determined for identifying this species in the future. DNA extraction and PCR amplification are described in Karasawa and Honda (2012). The primers and the accession numbers are shown in Tables 1 and 2, respectively.

**Table 1.** PCR primers used in this study.

Genes	Primer	Sequences (5' to 3')	Source
Forward			
COI	LCO1490	GGTCAACAAATCATAAAGATATTGG	Folmer et al. (1994)
12S	12Sai	AAACTAGGATTAGATACCCTATTAT	Palumbi (1996)
16S	16Sar-int-sf	GCCGCAGTATHCTRACTGTGCT	Parmakelis et al. (2008)
18S	18Sforward	TACCTGGTTGATCCTGCCAG	Maraun et al. (2009)
28S	D3A	GACCCGTCTTGAAACACGGA	Litvaitis et al. (1994)
Reverse			
COI	HCO2198	TAAACTTCAGGGTGACCAAAAAATCA	Folmer et al. (1994)
12S	12Sbi	AAGAGCGACGGGCGATGTGT	Palumbi (1996)
16S	16Sbr	CCGGTCTGAACTCAGATCACGT	Klossa-Kilia et al. (2006)
18S	18S614r	TCCAAC TACGAGCTTTTTAACC	Maraun et al. (2009)
28S	D3B	TCGGAAGGAACCAGCTACTA	Litvaitis et al. (1994)

**Table 2.** Locality, DDBJ accession numbers and voucher specimens of *Burmoniscus meeusei*.

Locality	DDBJ sccession no.					Voucher specimens
	COI	12S	16S	18S	28S	
Minami, Kitadaito Village, Kitadaitojima Island, Okinawa Prefecture, Japan	AB889795	AB889798	AB889801	AB889804	AB889807	KMNH-IvR 500720
Daitogu, Kitadaito Village, Kitadaitojima Island, Okinawa Prefecutre, Japan	AB889794	AB889797	AB889800	AB889803	AB889806	KMNH-IvR 500722
Yamato Village, Amamioshima Island, Kagoshima Prefecture, Japan	AB889796	AB889799	AB889802	AB889805	AB889808	KMNH-IvR 500723

## Results

### Genus *Burmoniscus* Collinge, 1914

#### Burmoniscus meeusei

(Holthuis, 1947)

http://species-id.net/wiki/Burmoniscus_meeusei

Figs 1 – 3

Chaetophiloscia meeusei Holthuis, 1947: p.124–130, Figs 1–2.
Brumoniscus meeusei : Taiti and Ferrara 1991, p.212, Figs 7–8; Kwon and Jeon 1993, p.142–143, Fig. 7; Araujo et al. 1996, p.118–120, Figs 15–21.
Brumoniscus kitadaitoensis Nunomura, 2009, p.79–81, Fig. 3; Nunomura 2011, p.60. Syn. n.

##### Material examined.

Holotype of *Burmoniscus kitadaitoensis*, TOYA-Cr-14899, male, dissected, near Daitogu, Kitadaitojima Island, Okinawa Prefecture, Japan, 25th November 2006, Noboru Nunomura leg; non types, 2 male, KMNH-IvR 500720 and 500721, 25.9314°N, 131.3094°E, Minami, Kitadaito Village, Kitadaitojima Island, Okinawa Prefecture, Japan, 30th June 2012, Takeshi Goto leg.; non type, 1 male, KMNH-IvR 500722, 25.9444°N, 131.3021°E, Daitogu, Kitadaito Village, Kitadaitojima Island, Okinawa Prefecture, Japan, 30th June 2012, Takeshi Goto leg.; non type, 1 male, KMNH-IvR 500723, 28.3560°N, 129.3935°E, Yamato Village, Amamioshima Island, Kagoshima Prefecture, Japan, 12th September 2012, Shigenori Karasawa leg.

##### Remarks.

**Figure 1.**
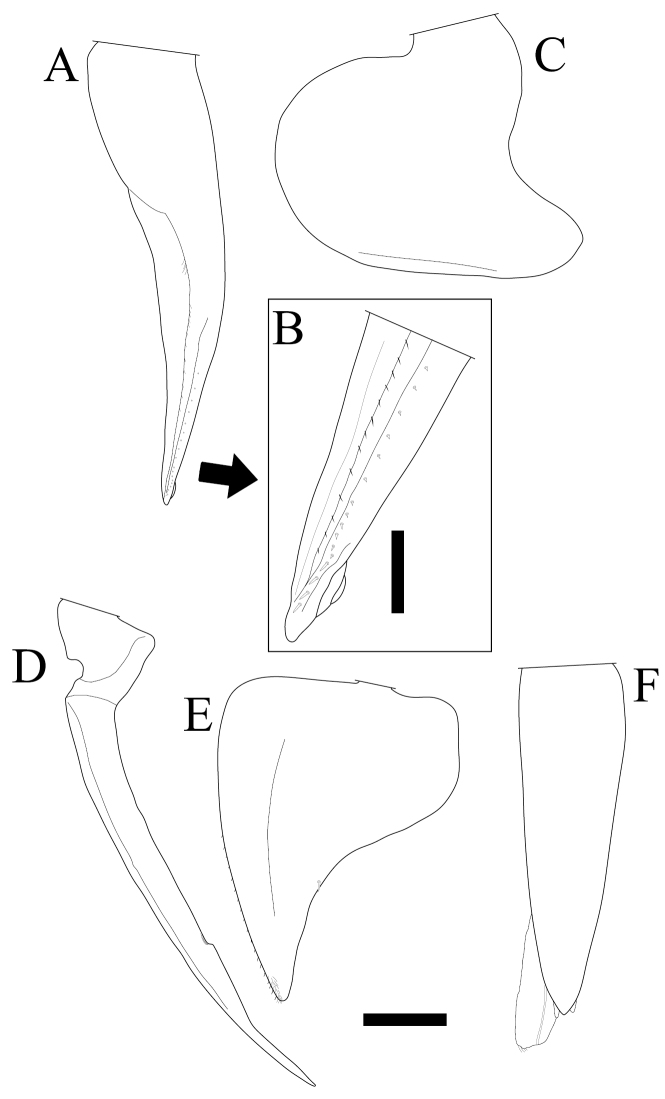
*Burmoniscus kitadaitoensis*, male, holotype, TOYA-Cr 14899. **A, B** Pleopod 1 endopodite **C** pleopod 1 exopodite **D** pleopod 2 endopodite **E** pleopod 2 exopodite **F** genital papilla. Scale bars: **A, C–E** 200 μm, **B** 50 μm.

**Figure 2.**
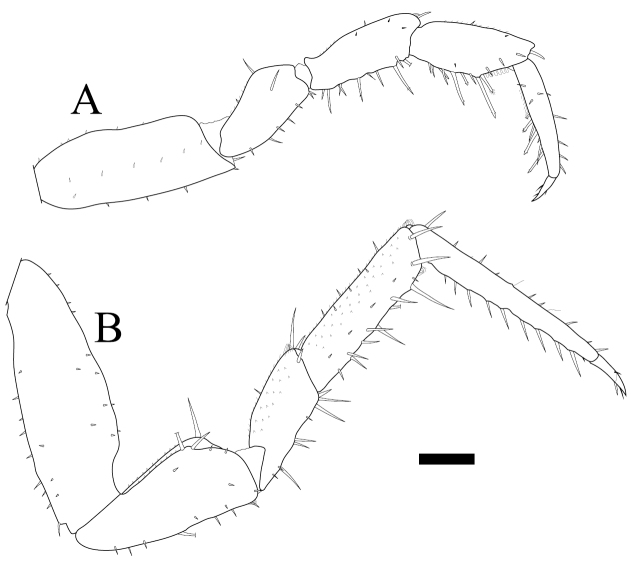
*Burmoniscus kitadaitoensis*, male, holotype, TOYA-Cr 14899. **A** Pereiopod 1 **B** pereiopod 7. Scale bar: 200 μm.

**Figure 3.**
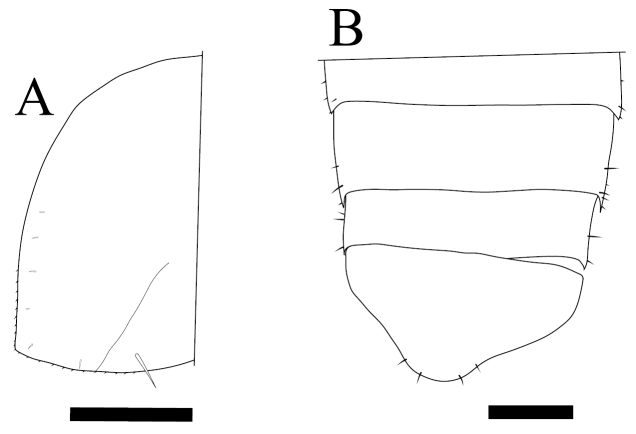
*Burmoniscus kitadaitoensis*, male, holotype, TOYA-Cr 14899. **A** Left epimeron of pereonite 7 **B** telson. Scale bars: 300 μm.

*Burmoniscus meeusei* is characterized by the male pleopod 1 endopodite slender and gradually narrowing to the apex (Fig. 1A) with a small lobe on the inner margin close to the apex (Fig. 1B); the male pleopod 1 exopodite with triangular posterior point bent outward and inner margin evenly convex (Fig. 1C); the male pleopod 2 endopodite slender (Fig. 1D) and exopodite trapezoidal (Fig. 1E); genital papilla elongated and simple (Fig. 1F); male pereiopods 1 and 7 without particular modifications (Fig. 2A, B); the pereonite 7 with postero-lateral corners at obtuse angle (Fig. 3A); and the apex of pleotelson broadly rounded (Fig. 3B).

**Figure 4.**
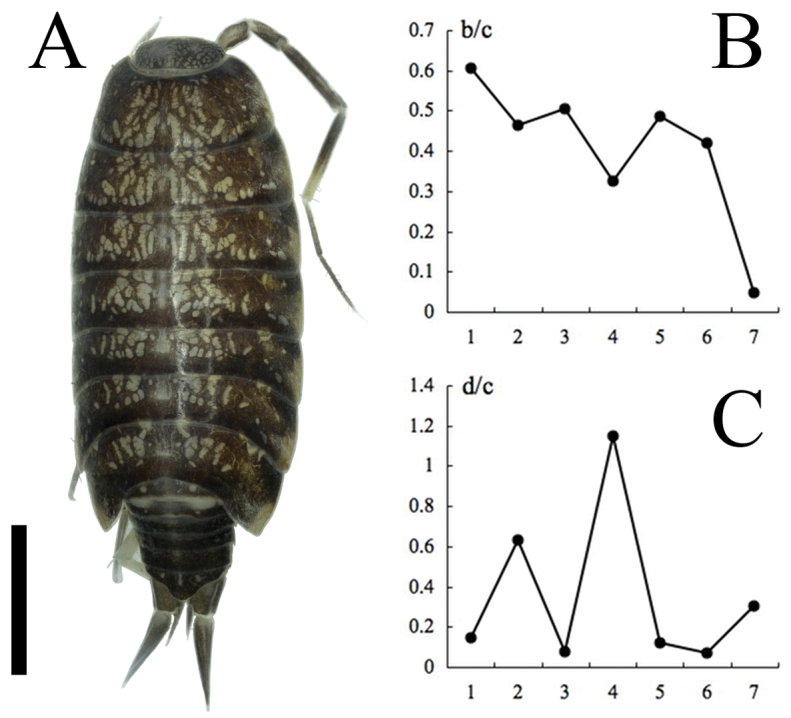
*Burmoniscus meeusei*, male, collected from Kitadaitojima Island, Japan. **A** Dorsal view of body, left antenna 2 broken, KMNH-IvR 500720 **B** co-ordinate of noduli laterales (b/c), KMNH-IvR 500721 **C** co-ordinate of noduli laterales (d/c), KMNH-IvR 500721. Scale bar: 1 mm.

On the holotype of *Burmoniscus kitadaitoensis*, a small lobe was found on the inner margin of the male pleopod 1 endopodite, although this character was not shown in the original description (Fig. 3P in Nunomura 2009). Moreover, the other morphological characters including the co-ordinate of the noduli laterales (Fig. 4) are consistent with those of *Burmoniscus meeusei* (see Figs 1 and 2 in Holthuis 1947; Figs 7 and 8 in Taiti and Ferrara 1991; Fig. 7 in Kwon and Jeon 1993; Figs 15–21 in Araujo et al. 1996). Thus the present study considers *Burmoniscus kitadaitoensis* as a junior synonym of *Burmoniscus meeusei*.

##### Distribution.

*Burmoniscus meeusei* was previously reported from the United Kingdom (greenhouses), Hawaii, Brazil, Taiwan (Schmalfuss 2004) and Japan. In Japan this species has been collected from Kitadaitojima Island only (Nunomura 2009, 2011), but the present study found the species on Amamioshima Island, which is about 300 km from Kitadaitojima Island.

##### DNA sequences.

The COI, 12S rRNA, 16S rRNA, 18S rRNA and 28S rRNA alignments comprised 653, 354, 453, 675 and 635 bp, respectively. With the exception of the 12S rRNA gene, there is no difference in the four genes among the specimens collected from Kitadaitojima and Amamioshima Islands. Only one 12S rRNA gene base varied between the specimens collected from the two islands.

## Supplementary Material

XML Treatment for Burmoniscus meeusei
